# Nitric Oxide as a Beneficial Signaling Molecule in *Trichoderma atroviride* TRS25-Induced Systemic Defense Responses of Cucumber Plants Against *Rhizoctonia solani*

**DOI:** 10.3389/fpls.2019.00421

**Published:** 2019-04-17

**Authors:** Justyna Nawrocka, Aleksandra Gromek, Urszula Małolepsza

**Affiliations:** Department of Plant Physiology and Biochemistry, Faculty of Biology and Environmental Protection, University of Lodz, Lodz, Poland

**Keywords:** *Trichoderma*, *Rhizoctonia solani*, plant defense responses, signaling, nitric oxide, salicylates

## Abstract

In the present study, *Trichoderma atroviride* TRS25 is presented as a biological control agent, which significantly limits the development of infection and reduces the disease caused by the pathogenic fungus *Rhizoctonia solani* in cucumber plants (*Cucumis sativus* L.). The systemic disease suppression is related to oxidative, signaling, and biochemical changes, that are triggered in response to a pathogen. Induction of systemic defense in cucumber by TRS25 greatly depends on the accumulation of signaling molecules including hydrogen peroxide (H_2_O_2_) and nitric oxide (NO) as well as salicylic acid (SA) and its derivatives including methyl salicylate (MeSA) and octyl salicylate (OSA). The study established that NO was accumulated in leaves and shoots of the cucumber plants, especially those pretreated with *Trichoderma* and inoculated with *R. solani*, where the compound was accumulated mainly in the cells localized in the vascular bundles and in epidermal tissues. We suggest, for the first time, that in the plants pretreated with TRS25, the accumulation of H_2_O_2_ and NO may be related to catalase (CAT) and S-nitrosoglutathione reductase (GSNOR) activity decrease. On the other hand, excessive accumulation of NO and SA may be controlled by forming their inactive forms, S-nitrosothiols (SNO) and salicylic acid glucosylated conjugates (SAGC), respectively. The obtained results suggest that the mentioned molecules may be an important component of the complex signaling network activated by TRS25, which is positively involved in systemic defense responses of cucumber plants against *R. solani*.

## Introduction

*Trichoderma* spp. are the most common saprophytic fungi in the rhizosphere and they are very effective biological control agents (BCAs) impairing pathogens with different antagonistic strategies. Antibiosis, mycoparasitism, competition for niches and nutrients as well as induction of local and systemic defense responses in plants are suggested as the mechanisms by which *Trichoderma* controls infection process and disease development (Harman et al., 2004; Vinale et al., 2008; Elsharkawy et al., 2013; Gomes et al., 2017). Induction of plant defense mechanisms or “sensitization” of plants by prior application of *Trichoderma* is thought to be a promising plant protection strategy. However, their beneficial influence on plants depends on the *Trichoderma* strain, plant species, pathogen, and soil-climate conditions (Harman et al., 2004; Singh et al., 2010; Hermosa et al., 2012; Yousef et al., 2013; Gomes et al., 2017).

Induced resistance is associated with stimulation of the plant immune system by elicitors, molecules that mimic pathogen attack, or by living organisms. Different responses are induced in plants after detection and recognition of elicitor molecules (Pieterse et al., 2014; Kushalappa et al., 2016; Oliveira et al., 2016). The perception of *Trichoderma* elicitors by plants involves various cellular and molecular host-defense responses, for example, enhanced generation of reactive oxygen species (ROS) and activation of defense-related gene expression. Moreover, *Trichoderma* may induce accumulation of antimicrobial compounds such as phenolics as well as it may cause cell wall reinforcement by lignin and callose deposition (Bolton, 2009; Salas-Marina et al., 2011; Harman et al., 2012; Mayo et al., 2015).

The induced defense responses are often linked to the activation of a complex signal transduction network, which involves mainly salicylic acid (SA), jasmonic acid (JA), with or without ethylene (ET) and abscisic acid (ABA), as important regulators of plant immunity, depending on the pathosystem (Contreras-Cornejo et al., 2011; Hermosa et al., 2012; Sivakumaran et al., 2016; Checker et al., 2018). Phytohormones are found to play a crucial regulatory role in two main types of induced resistance: systemic acquired resistance (SAR) with SA-mediated signaling pathway leading to resistance to biotrophic and hemibiotrophic pathogens, and induced systemic resistance (ISR) in which JA/ET regulate defense against necrotrophic pathogens (Pieterse et al., 2014). In plants, *Trichoderma* is able to activate defense mechanisms leading to ISR, rarely SAR, and according to the results of the latest studies, to mixed ISR/SAR type of resistance called *Trichoderma*-induced systemic resistance (TISR) (Martínez-Medina et al., 2013; Leonetti et al., 2017). Overlapping signaling pathways which involve both SA and JA/ET-mediated signal transduction pathways may induce defense reactions against both biotrophic and necrotrophic pathogens, thus increasing the resistance of the plants (Martínez-Medina et al., 2013; Pieterse et al., 2014). The mechanism of defense responses induction and the type of resistance induced in plants by *Trichoderma* still remain controversial (Vinale et al., 2008; Harman et al., 2012). There are many gaps and divergences in the physiological, biochemical, and molecular changes related to *Trichoderma*-induced defense responses providing resistance in plants, especially when it comes to explaining crosstalk of signaling molecules. In this instance, in order to elucidate crucial interaction of JA, ET, SA, and their derivatives in the complex signaling network, special attention should be paid to other compounds which may down- or upregulate their synthesis and activity in different ways. Recent studies have indicated that plants also make use of redox-active small molecules including ROS and reactive nitrogen species (RNS) as messengers for the management of crosstalk between secondary messengers, hormones, and mitogen-activated protein kinases (MAPKs), important in plant resistance development. Among all ROS and RNS, there is increasing focus on hydrogen peroxide (H_2_O_2_) and nitric oxide (NO) as the molecules with high potential to interact with various hormonal signaling pathways creating so called oxidative signaling (Tada et al., 2008; del Río, 2015; Weidinger and Kozlov, 2015). NO was shown to be rapidly generated in plants following a challenge with biotrophic and necrotrophic pathogens and was considered as one of the major defense activators (Mur et al., 2013). The mechanisms through which NO might impact defense signaling cascades were extensively examined. The reversible S-nitrosylation of proteins, that is, the reaction of NO with cysteine-rich thiol groups in proteins to form S-nitrosothiols (SNO) (Spadaro et al., 2010) as well as nitration of tyrosine-rich groups in proteins, emerged as important regulatory events determined by NO. Modification of the activity of the SA signaling component, NPR1 protein (Tada et al., 2008) and of the ROS generating complex NADPH oxidase, AtRBOHD (Yun et al., 2011; Sivakumaran et al., 2016) are notable examples of the role of S-nitrosylation in defense responses. There is clear evidence that NO may work together with ROS and SA to establish SAR (Gao et al., 2014; Mittler and Blumwald, 2015; Sami et al., 2018). On the other hand, the close interaction of NO with other signaling pathways of induced resistance which also include JA and ET was observed as well (Mur et al., 2013; Sami et al., 2018). The essential role of NO in plant signaling networks has been widely observed in different plant species under various abiotic and biotic stress conditions, such as viral diseases and bacterial or fungal infections. However, the role of NO in *Trichoderma*-induced plant defense responses against pathogen infection and the relationship between NO signaling and TISR has not been studied so far.

Cucumber plants, *Trichoderma atroviride* TRS25 as biological control agent (BCA) and the pathogen *Rhizoctonia solani* Kühn were used in the present study. In the preliminary studies, TRS25 was chosen from 25 *Trichoderma* isolates, as the most effective in cucumber growth and development promotion and yielding enhancement as well as reduction of the incidence of *R. solani* disease (Nawrocka et al., 2011). The applied pathogen *R. solani* is still regarded as one of the most destructive ones for greenhouse and field-grown cucumbers in which it causes root and shoot rot or foliar blight. As the use of chemicals and changes in the cultivation practices are not sufficiently effective in the protection of plants against *R. solani*, the induction of defense responses and resistance to the pathogen by *Trichoderma* has emerged as potential supplement of crop protection (Singh et al., 2002; Bartz et al., 2010; Saberi et al., 2013; Yousef et al., 2013; Anderson et al., 2017). Firstly, some *Trichoderma* strains were listed among the effective antagonistic or mycoparasitic BCA of *R. solani* (Harman et al., 2004; Yousef et al., 2013). Further analyses suggested that resistance induction together with overexpression of different pathogenesis related proteins (PR) might be one of the ways of plant protection against *R. solani* as well. However, this process requires further elucidation (Yousef et al., 2013; Mayo et al., 2015). Because our previous results strongly pointed to *T. atroviride* TRS25 as BCA of *R. solani*, we decided to study the signaling network involved in the effective cucumber defense against this pathogen, induced by TRS25. Based on the preliminary studies we hypothesized that in cucumber plants TRS25 might induce a complex signaling network of ROS, NOS, and salicylates, which trigger defensive reactions providing plant protection similar to TISR. Therefore, the primary aim of the present study was to investigate the molecules that play an important role in the complex signaling network induced by TRS25 in cucumber plants against *R. solani*. Special attention was paid to the changes in the content of ROS, RNS, SNO, and salicylates together with the changes in the activity of antioxidant enzymes, i.e., superoxide dismutase (SOD), catalase (CAT) as well as S-nitrosoglutathione reductase (GSNOR). To the best of our knowledge, these compounds and enzymes have not been studied in relation to the protection of cucumber plants against *R. solani* induced by *Trichoderma.*

## Materials and Methods

### The Growth of *Trichoderma atroviride* and *Rhizoctonia solani* and Inoculum Preparation

*T. atroviride* TRS25 was obtained from the Research Institute of Horticulture (Skierniewice, Poland). The morphological identification and molecular classification of TRS25 were described previously by Oskiera et al. (2015) and deposited at the NCBI GenBank with accession numbers: ITS KJ786731 and tef1α KJ786812. The complementary tests of antagonistic or mycoparasitic properties of TRS25 isolate against various pathogens including *R. solani* showed moderate ability of this strain to colonize, overgrow, or parasitize fungal sclerotia (Szczech et al., 2014). Before use for cucumber pretreatment, the TRS25 strain was grown on malt extract agar medium (Fluka) for 10 days at 25°C. To obtain TRS25 inoculum, the spores of the fungus were washed off the surface with 0.85% NaCl solution and adjusted to 10^6^ cfu/cm^3^. The spore suspension was added to the growing substrate before the sowing of cucumber seeds.

*R. solani* Kühn MUCL47938 strain obtained from the Research Institute of Horticulture (Skierniewice, Poland), used in the experiment, is a well-characterized, standard phytopathogen of cucumber plants. Before cucumber plants inoculation, the fungus was grown for 7 days in 9-cm diameter Petri plates on potato dextrose agar, in the dark, at 25°C. After incubation, mycelial mats of five plates were homogenized in 0.5 L of deionized water and used to inoculate the cucumber plants.

### Plant Material, Growth Conditions, and Procedure of Treatment With Microorganisms

Four-week-old cucumber plants (*Cucumis sativus* L.) cv. Iwa F1, susceptible to *R. solani*, were used in the experiment. The plants were cultivated in pots (one seed per pot) with podsolic soil and vermiculite 1:1 (v:v) and grown in a chamber at a temperature of 25/20°C with 16/8 h day/night photoperiod at 70% relative humidity. Light was supplied by white fluorescent lamps (type 36 W, Philips TDL 36/84) at 350 μEm^−2^ s^−1^ intensity. The growing substrate of half of the plants was supplemented with aliquots of the *T. atroviride* TRS25 spore suspension to obtain 10^6^ spore density per 1 g of the substrate. The substrate without TRS25 was used to grow control plants. One week before biochemical analyses, half of the control and half of the TRS25 pretreated plants were inoculated with 5 ml of *R. solani* mycelium homogenate around the cucumber stem base, according to the method presented by Pannecoucque et al. (2008). Four experimental groups of plants were tested: (I) control plants, (II) *Rs* plants – non treated with TRS25 and inoculated with *R. solani*, (III) TRS25 plants – pretreated with TRS25, uninoculated with *R. solani*, and (IV) TRS25 + Rs plants – pretreated with TRS25 and inoculated with *R. solani*. The disease area on the roots and leaves of cucumber plants was evaluated using a Motic Images Plus 2.0ML program (Motic China Group, Asia) according to the manufacturer’s instruction. Disease severity was scored using the following scale: 0 = no lesions and rot symptoms; 1 = lesions and rot symptoms up to 25% of leaf or root area; 2 = lesions and rot symptoms from 25 to 50% of leaf or root area; 3 = lesions and rot symptoms from 50 to 75% of leaf or root area; and 4 = lesions and rot symptoms more than 75% of leaf or root area. Then, a disease index (DI) was calculated separately for each variant according to the formula described by Taheri and Tarighi (2010), where DI = [(1*n*_1_ + 2*n*_2_ + 3*n*_3_ + 4*n*_4_)/4*N*] × 100%, with *n*_1_ as the number of plants with score 1, *n*_2_ as the number of plants with score 2, etc., and *N* as the total number of plants used in the variant. For biochemical assays, the third leaf of each plant was cutoff. The experiment was prepared five times under the same conditions. In each experiment, the samples for biochemical analyses were prepared in triplicate per variant.

### Assay of Superoxide ($O_{2}^{.-}$) Content and Histochemical Visualization

Determination of $O_{2}^{.-}$ content was performed according to the modified method of Doke (1983). The cucumber leaves were cut into five 9-mm diameter discs and incubated for 1 h in 3 ml of 50 mM potassium phosphate buffer, pH 7.8 containing 0.05% staining nitroblue tetrazolium (NBT) (Sigma-Aldrich), 0.1 mM EDTA, and 0.065% NaN_3_ at room temperature (RT). Then the leaves were removed, the mixtures were heated at 85°C for 15 min and subsequently cooled. The $O_{2}^{.-}$ content was determined indirectly by measurement of an increase in the absorbance (A) at 580 nm related to NBT reduction, and expressed as A_580_ g^−1^ of fresh weight (FW). Histochemical visualization of $O_{2}^{.-}$ was performed according to the modified method of Romero-Puertas et al. (2004). Leaf pieces of the cucumber plants were cut and incubated with a solution of 0.05% NBT in 50 mM potassium phosphate buffer, pH 7.8, containing 10 mM NaN_3_, for 8 h in the dark and at RT, then subsequently rinsed and discolored with 95% ethanol in 70°C, for 10 min, under vacuum. This treatment decolorized the leaf pieces except for the dark blue insoluble precipitate resulting from the reduction of NBT in the presence of $O_{2}^{.-}$. After cooling, the leaves were extracted at RT with fresh ethanol for 4 h, then preserved at RT in ethanol and photographed.

### Assay of Hydrogen Peroxide (H_2_O_2_) Content and Histochemical Visualization

Determination of H_2_O_2_ content was performed according to the modified method of Capaldi and Taylor (1983). Leaf samples (500 mg) were homogenized in a mortar with liquid nitrogen and then with 2.5 ml 5% trichloroacetic acid (TCA) and 50 mg of activated charcoal. The homogenate was filtered, centrifuged (20,000 × *g*, 20 min) and the obtained supernatant was adjusted to pH 3.6 with 4 M KOH. The reaction mixture contained 0.2 ml of the leaf extract and 0.1 ml of 3.4 mM 3-methylbenzothiazoline hydrazone (MBTH). The reaction was initiated by adding 0.5 ml of horseradish peroxidase solution (90 U per 100 ml) in 0.2 M sodium acetate (pH 3.6) and ended by adding 1 ml of 1 N HCl. Absorbance related to oxidative coupling MBTH in the presence of H_2_O_2_ was read at 630 nm after 15 min. H_2_O_2_ content was calculated based on the standard curve of H_2_O_2_ and expressed in μmol per g of FW. Histochemical visualization of H_2_O_2_ was performed according to the modified method of Thordal-Christensen et al. (1997). Leaf pieces of the cucumber plants were cut and incubated with a solution of 1 mg cm^−3^ 3,3′-diaminobenzidine-tetrahydrochloride stain (DAB) (Sigma-Aldrich) in 50 mM acetate buffer, pH 3.8 for 8 h in the light and at RT, then subsequently rinsed and discolored with 95% ethanol in 70°C, for 10 min, under vacuum. This treatment decolorized the leaf pieces except for the brown polymerization product resulting from the reaction of H_2_O_2_ with DAB. After cooling, the leaves were extracted at RT with fresh ethanol for 4 h, then preserved at RT in ethanol, and photographed.

### Assay of Nitric Oxide (NO) Content and Histochemical Visualization

Determination of NO content was performed according to the modified method of Ding et al. (1988). Leaf samples (400 mg) were homogenized in a mortar with liquid nitrogen and then with 2 ml 50 mM acetic acid buffer, pH 3.6, containing 4% zinc diacetate. After centrifugation (20,000 × *g*, 20 min), the supernatant was collected and adjusted to pH 7.0 with 4 M KOH. The reaction mixture contained the leaf extract and its equivalent volume of Griess reagent (Sigma-Aldrich). Absorbance related to reaction of NO ions with Griess reagent to form a chromophoric azo product that absorbs strongly at 540 nm was determined after 30-min incubation of the mixture at RT. Indirectly, NO content was calculated by comparison to a standard curve using NaNO_2_ and expressed in nmol per g of FW. Histochemical visualization of NO was performed according to the modified method of Corpas et al. (2004) and Piterková et al. (2009). 100 μm-thick sections of the cucumber leaves, stems, and roots were cut using vibratome, then subsequently immersed and incubated for 30 min at RT in the dark, in 10 mM Tris–HCl buffer, pH 7.0 containing 10 μM 4,5-diaminofluorescein diacetate (DAF-2DA). Then the sections were washed two times in fresh buffer to wash off excess fluorophore, mounted in buffer on microscope slides, and examined immediately under confocal laser scanning microscope system (Leica TCS SP8; Leica Microsystems, Mannheim, Germany), using standard filters and collection modalities for DAF-2 green fluorescence (excitation 488 nm; emission 530 nm). The production of green fluorescence under the presented conditions was attributed to the presence of NO. As negative controls, before staining with DAF-2DA, some leaf pieces were immersed for 30 min in the dark, in 10 mM Tris-HCl buffer, pH 7.0 containing 0.1 mM 2-(4-carboxyphenyl)-4,4,5,5-tetramethylimidazoline-1-oxyl-3-oxide (cPTIO) (Sigma-Aldrich), which eliminate NO by production of nitrites. The slides were scanned using Leica LAS-AF program, version 3.3.0.

### Assay of S-Nitrosothiols (SNO) Content

Determination of SNO content was performed according to the modified method of Rustérucci et al. (2007). Leaf samples (400 mg) were homogenized in a mortar with liquid nitrogen, then with 2 ml 100 mM Tris-HCl buffer, pH 6.8, and centrifuged (15,000 × *g*, 15 min). The extracts were incubated for 5 min separately with an equivalent volume of solution A (1% sulfanilamide dissolved in 0.5 M HCl) or of solution B (solution A plus 0.2% HgCl_2_) which by hydrolyzing SNO allow to form diazonium salt. After 5 min, an equivalent volume of solution C [0.02% N-(1-naphthyl)ethylenediamine dihydrochloride dissolved in 0.5 M HCl] was added both to samples with A and B reagents. Absorbance related to formation of the azo dye product, obtained through reaction of diazonium salts with reagent C, was read at 550 nm after 5 min incubation. SNO were quantified on the basis of the difference of absorbance between samples with solution B and appropriate blank solution (A) (B − A), comparing the values with a standard curve made from the solution of S-nitrosoglutathione (GSNO) (Sigma-Aldrich). The results were expressed in nmol SNO per mg of protein.

### Assay of Superoxide Dismutase (SOD, EC 1.15.1.1) Activity

Determination of SOD activity was performed following the method of Beauchamp and Fridovich (1971). Leaf samples (400 mg) were homogenized in a mortar with liquid nitrogen and then with 2 ml 50 mM potassium phosphate buffer, pH 7.0, containing 0.5 M NaCl, 1 mM EDTA, and 1% polyvinylpyrrolidone (PVP), and then centrifuged (15,000 × *g*, 15 min). Analysis of SOD activity was based on monitoring its ability to inhibit the photochemical reduction of NBT. The incubation mixture contained 50 mM potassium phosphate buffer pH 7.8, 13 mM methionine, 75 μM NBT, 2 μM riboflavin, 0.1 mM EDTA, and the enzyme extract. The reaction was started by irradiation with ultraviolet (UV) light. The absorbance was measured after 10 min at 560 nm. SOD ability to inhibit 50% of the photochemical reduction of NBT as compared to the control was used as one unit (U) of its activity normalized per mg of protein.

### Assay of Catalase (CAT, EC.1.11.1.6) Activity

Determination of CAT activity was performed following the method of Dhindsa et al. (1980). Leaf samples (500 mg) were ground in a mortar with liquid nitrogen and then with 2.5 ml 50 mM sodium phosphate buffer, pH 7.0, containing 1 mM EDTA, 1 mM sodium ascorbate, and 0.5 M NaCl, and then centrifuged (15,000 × *g*, 15 min). The reaction mixture contained 50 mM potassium phosphate buffer (pH 7.0), enzyme extract, and 15 mM H_2_O_2_ starting the reaction. The consumption of H_2_O_2_ by CAT was monitored spectrophotometrically at 240 nm. CAT activity was calculated on the basis of the millimolar extinction coefficient of H_2_O_2_ ε = 45.2 mM cm^−1^ and presented in enzymatic units (U) of H_2_O_2_ decomposed min^−1^ per mg of protein.

### Assay of S-Nitrosoglutathione Reductase (GSNOR, EC.1.1.1.284) Activity

Determination of GSNOR activity was performed following the method of Sakamoto et al. (2002). Leaf samples (400 mg) were homogenized in a mortar with liquid nitrogen and then with 2 ml 50 mM Tris-HCl buffer, pH 8.0, containing 0.1% Tween 20, and then centrifuged (15,000 × *g*, 15 min). Analysis of GSNOR activity was based on monitoring its ability to reduce GSNO by using NADH. The incubation mixture contained 20 mM Tris-HCl buffer pH 8.0, 200 μM NADH, GSNO with a final concentration of 400 μM and the enzyme extract. The absorbance was measured for 4 min at 340 nm. GSNOR enzyme activity was calculated on the basis of the millimolar extinction coefficient of NADH *ε* = 6.22 mM cm^−1^ at 340 nm and presented in enzymatic units (U) of NADH min^−1^ per mg of protein.

### Assay of Protein Content

Protein was determined by the method of Bradford (1976) with standard curves prepared using bovine serum albumin (Sigma-Aldrich).

### Assay of Salicylate Content

Total salicylic acid (total SA) and SA glycosylated conjugates (SAGC) were extracted according to the modified protocol of Molina et al. (2002). Leaf samples (1 g) were extracted three times in 10 ml of 80, 90, and then 100% MeOH. After centrifugation (15 min, 20,000 × g), all the supernatants were combined and evaporated to dryness under a vacuum at 65°C. The residue was re-dissolved in water at 80°C and centrifuged (15 min, 20,000 × g). Then total SA was extracted three times into three volumes of cyclopentane:ethylacetate:2-propanol (50:50:1, v/v/v). The organic extracts were dried under vacuum and resuspended in 70% MeOH containing 0.5% fumaric acid. To release SA from SAGC, the aqueous phase was acidified with HCl to pH 1.5–2.0 and boiled for 1.5 h at 80°C. The released SA was extracted with the organic mixture as described above. HPLC system (DIONEX, Sunnyvale, CA, USA) was used for analysis. Separation of SA took place over an RP column (aQ Hypersil GOLD, 250 mm × 4.6 mm, 5 μm) joined with a guard column (GOLD aQ Drop-In guards, 10 mm × 4 mm, 5 μm) at 40°C, by use of a binary solvent system consisting of (A) water and (B) methanol with 0.5% FA with a flow rate of 1.5 ml/min. The elution profile was as follows: 0–2 min, 40% B; 2–10 min, 40–60% B; 10–12 min, 60% B; 12–13 min, 60–40% B; and 13–15 min, 40% B. Chromatograms were obtained by fluorescence evaluation (excitation 301 nm, emission 412 nm). Quantification was based on the calibration curves for the adequate SA standards (Sigma-Aldrich).

Methyl salicylate (MeSA) and octyl salicylate (OSA) volatiles were isolated with the use of solid-phase microextraction (SPME) according to the method of Carlin et al. (2016). Freshly harvested leaves (3 g) were incubated in 20 ml headspace vials at 40°C for 30 min and subsequently extracted using 50/30 divinylbenzene/carboxen/polydimethylsiloxane (DVB/CAR/PDMS) 1 cm long fiber for 60 min. After that the samples were introduced into gas chromatograph injection port and desorbed at 240°C with a split ratio of 1:30. The samples were analyzed by GCxGC TOF-MS. The analysis was performed using Pegasus 4D mass spectrometer (LECO Corp.). A BPX5 (30 m, 0.25 mm, and 0.25 μm) was used as a first-dimension (1D) column, and a BPX50 (2 m, 0.1 mm, and 0.1 μm) was used as a second-dimension (2D) column with helium, constant flow 1 ml/min as a carrier gas. Temperature programme conditions were as follows: first oven +50°C (3 min) −280°C at 4°C/min, second oven and modulator, respectively, +5 and +20°C relatively to the first oven programme (modulation time 8 s, hot pulse time 2.4 s, cold pulse −80°C, time 1.6 s). TOF mass spectrometer parameters included mass range of m/z 33–550 at 30 spectra/s, ionisation energy of 70 eV, and ion source temperature of 200°C. Quantification was based on the calibration curves for the adequate SA standards (Sigma-Aldrich).

### Statistics

To determine the disease area as well as to analyze the biochemical parameters, the results from five independent, not significantly different trials, were combined. In all experiments, three replicates for each variant were obtained and sample variabilities were given as a standard deviation (SD) of the means. Statistical analysis of variance (ANOVA, *p* < 0.05) for each parameter was followed by the Duncan multiple range *post hoc* test. Respective significant differences were marked using different letters (a, b, c; A, B, C, etc.). All statistical estimates were performed using the Statistica 13.1 software.

## Results

### *T. atroviride* TRS25 Systemically Suppresses *R. solani* Infection of Cucumber Plants

**Figure 1:**
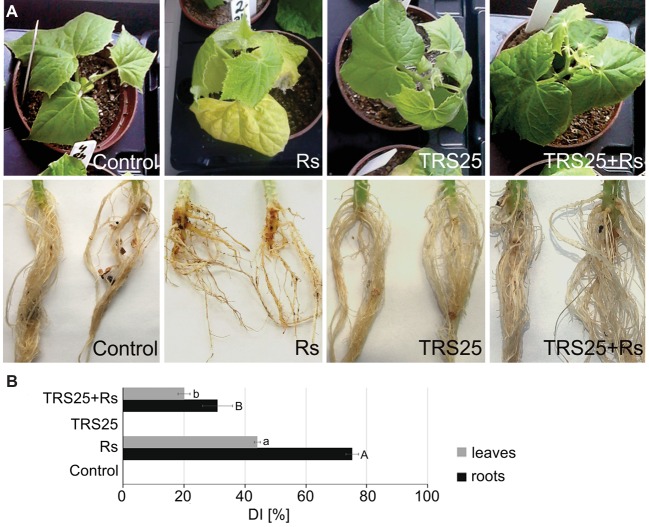
Evaluation of the disease symptoms, i.e., irregular brown lesions and rot symptoms on roots and shoots and dark brown blight blotches on shoots and  eaves, occurring on 5-week-old cucumber plants caused by the pathogenic fungus *R. solani* **(A)**. A disease index (DI) showing disease severity **(B)**. The values represent the means + SE from five independent experiments with three replicates per variant. Statistical analyses of variance (ANOVA, *p* < 0.05, Duncan multiple range *post hoc* test) performed separately for roots and leaves demonstrated significant differences at *p* ≤ 0.05 between variants marked by letters a and b for leaves and A and B for roots. Abbreviations: control (TRS25 nontreated uninoculated with *R. solani*), Rs (TRS25 nontreated, inoculated with *R. solani*), TRS25 (TRS25 pretreated uninoculated with *R. solani*), and TRS25 + Rs (TRS25 pretreated inoculated with *R. solani*).

To study whether TRS25 protects cucumber locally and systemically against *R. solani*, the disease symptoms were observed both on roots and leaves of plants growing in the substrate consisting *Trichoderma* and inoculated with *R. solani*. In general, successful infection of Rs plants by *R. solani* began within 4–5 days after inoculation and progressed rapidly. Disease symptoms were identified in all Rs plants based on the formation of extensive brown lesions and rot symptoms on the roots followed by shoot and leaf dark brown blight blotches and plant collapse (Figure 1A). The disease symptoms were significantly suppressed when spores of TRS25 were added to the plant growing substrate, as indicated by a significant reduction of the disease index (DI) in TRS25 + Rs plants within 7 days of pathogen inoculation. As compared to Rs plants, TRS25 caused pronounced DI decrease in the roots of TRS25 + Rs plants from 75 to 30% and in the leaves from 42 to 20% (Figure 1B).

### *T. atroviride* TRS25 Activates Systemic Biochemical Responses in Cucumber Plants

#### ROS Content

**Figure 2:**
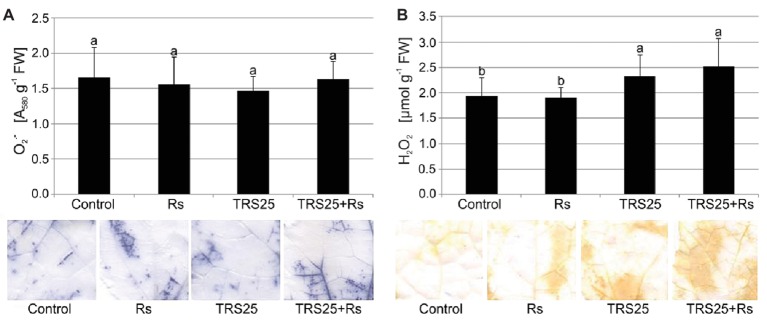
Effect of TRS25 on **(A)** superoxide ($O_{2}^{.-}$) and **(B)** hydrogen peroxide (H_2_O_2_) content in cucumber plants. Under the graphs, there are histochemical visualizations of compounds prepared with NBT for $O_{2}^{.-}$ detection (blue blotches) and with DAB for H_2_O_2_ detection (brown blotches). The number of replicates, calculations, and abbreviations is as in Figure 1. Statistical analyses of variance (ANOVA, *p* < 0.05, Duncan multiple range *post hoc* test) demonstrated significant differences between variants marked by letters a and b. Data points followed by a different letter are significantly different at *p* ≤ 0.05.

Biochemical analyses showed that *R. solani* inoculation did not cause significant changes in $O_{2}^{.-}$ and H_2_O_2_ content in Rs plants as compared to the control (Figures 2A,B). Simultaneously, in the same Rs plants, histochemical visualization showed no visible differences in $O_{2}^{.-}$ and H_2_O_2_ localization and the covered surface in leaves as compared to the control (Figures 2A,B). Pretreatment of cucumber plants with TRS25 did not significantly affect $O_{2}^{.-}$ content either in TRS25 or TRS25 + Rs plants as compared to the control and Rs plants (Figure 2A). On the other hand, biochemical analysis and histochemical visualization showed that the TRS25 strain significantly increased H_2_O_2_ content which covered larger leaf surface both in TRS25 and TRS25 + Rs plants as compared to the control and Rs plants (Figure 2B). The highest content of H_2_O_2_ was observed in TRS25 + Rs plants; however, it was not significantly different from that in TRS25 plants.

#### SOD and CAT Activities

**Figure 3:**
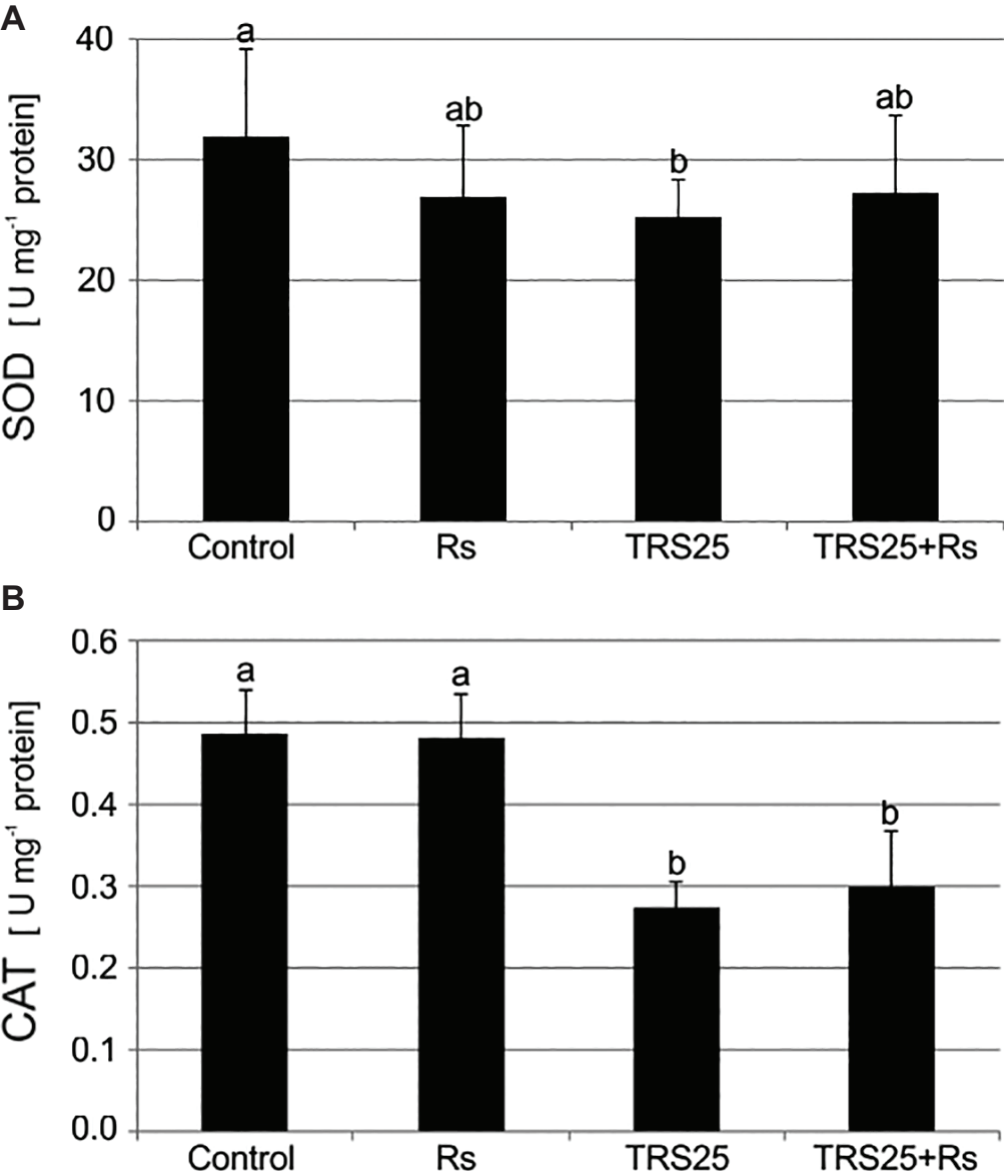
Effect of TRS25 on **(A)** superoxide dismutase (SOD) and **(B)** catalase (CAT) activity in cucumber plants. The number of replicates, calculations, and abbreviations is as in Figure 1. Statistical analyses of variance (ANOVA, *p* < 0.05, Duncan multiple range *post hoc* test) demonstrated significant differences between variants marked by letters a and b. Data points followed by a different letter are significantly different at *p* ≤ 0.05.

As compared to the control, *R. solani* did not affect SOD and CAT activities in Rs plants (Figures 3A,B). Simultaneously, the tested *Trichoderma* strain did not influence SOD activity in TRS25 + Rs plants and decreased it in TRS25 plants as compared to the control (Figure 3A). Moreover, no significant differences concerning SOD activity were observed between TRS25, TRS25 + Rs, and Rs plants (Figure 3A). In the case of CAT, strong suppression of this enzyme activity by 50 and 45%, respectively, as compared to the control and Rs plants, was detected both in TRS25 and TRS25 + Rs plants (Figure 3B). The activity of CAT was similar in TRS25 and TRS25 + Rs plants.

#### NO and SNO Contents

**Figure 4:**
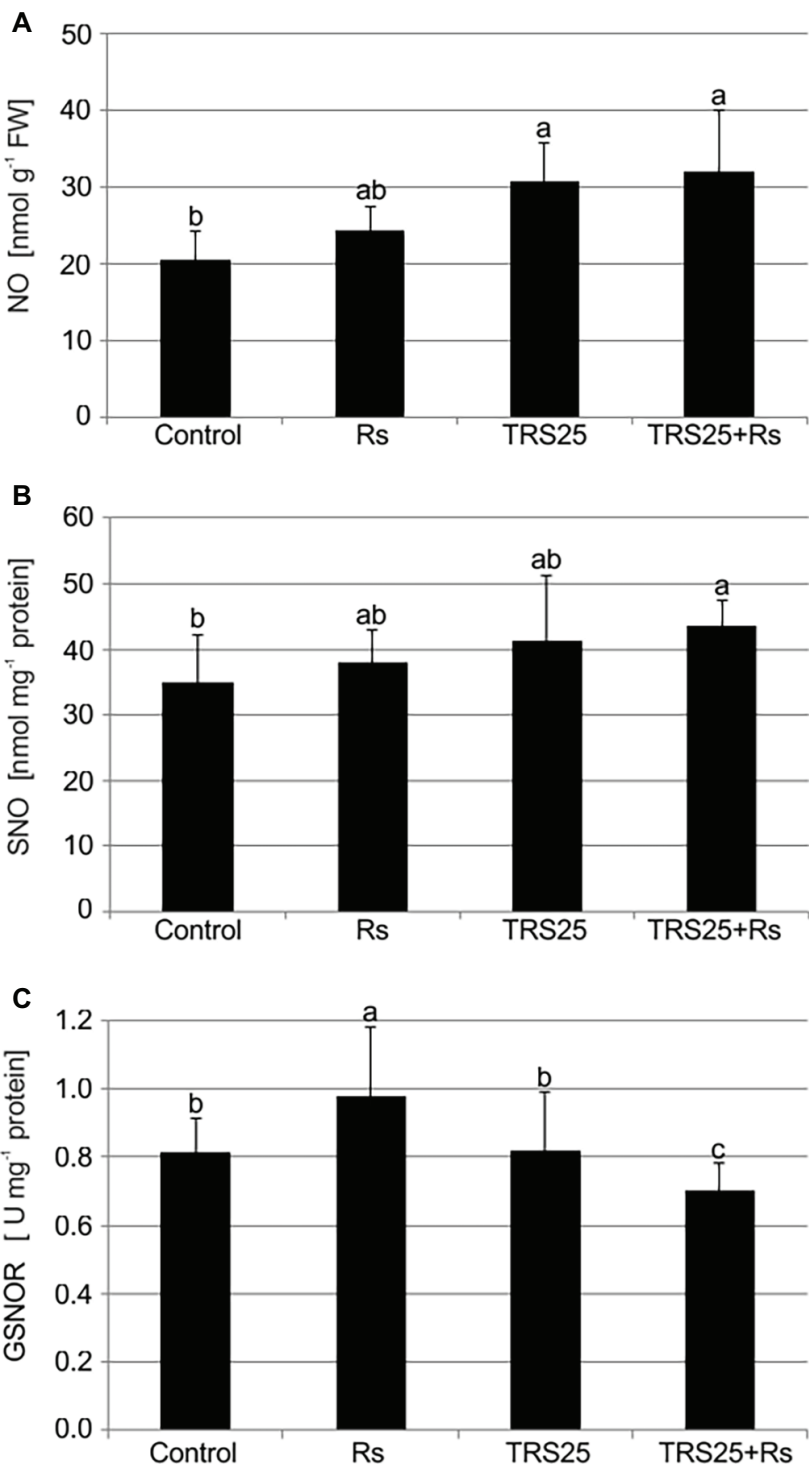
Effect of TRS25 on **(A)** nitric oxide (NO), **(B)** nitrosothiol (SNO) content, and **(C)** S-nitrosoglutathione reductase (GSNOR) activity in cucumber plants. The number of replicates, calculations, and abbreviations is as in Figure 1. Statistical analyses of variance (ANOVA, *p* < 0.05, and Duncan multiple range *post hoc* test) demonstrated significant differences between variants marked by letters a, b, and c. Data points followed by a different letter are significantly different at *p* ≤ 0.05.

**Figure 5:**
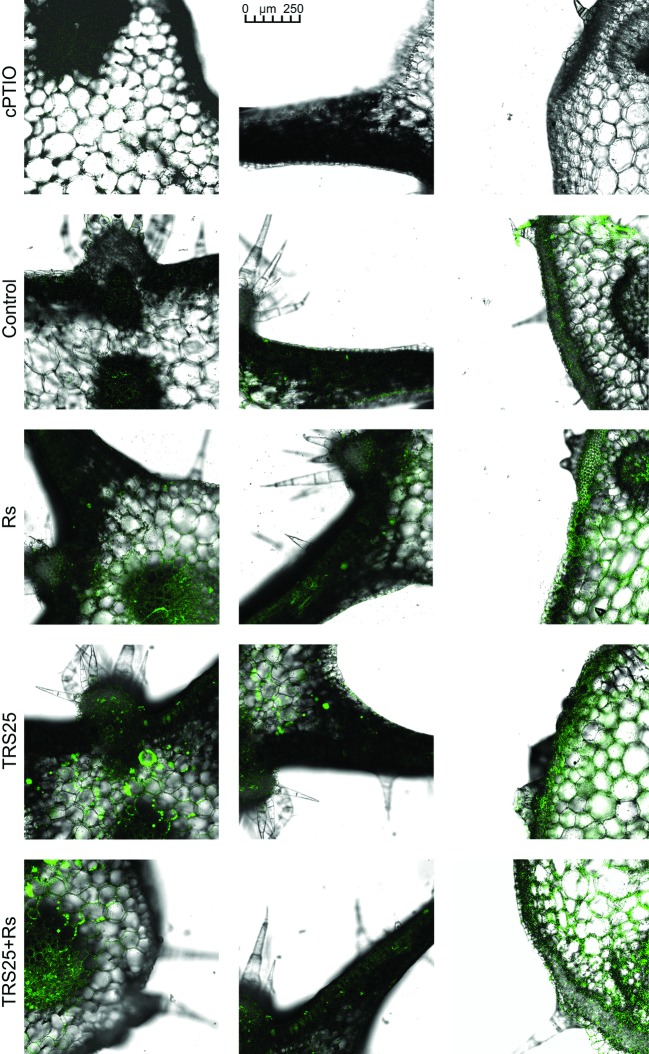
Cross sections of the cucumber leaves. Fluorescent confocal microscopy was used to detect NO localization in the cucumber plants. Light green fluorescence of NO was observed mainly in the cells localized in the vascular bundles as well as in the cells of epidermal tissues. DAF-2DA was used for histochemical visualization of NO. As negative control, leaf pieces were immersed in a buffer containing cPTIO which eliminates NO. Abbreviations are as in Figure 1.

**Figure 6:**
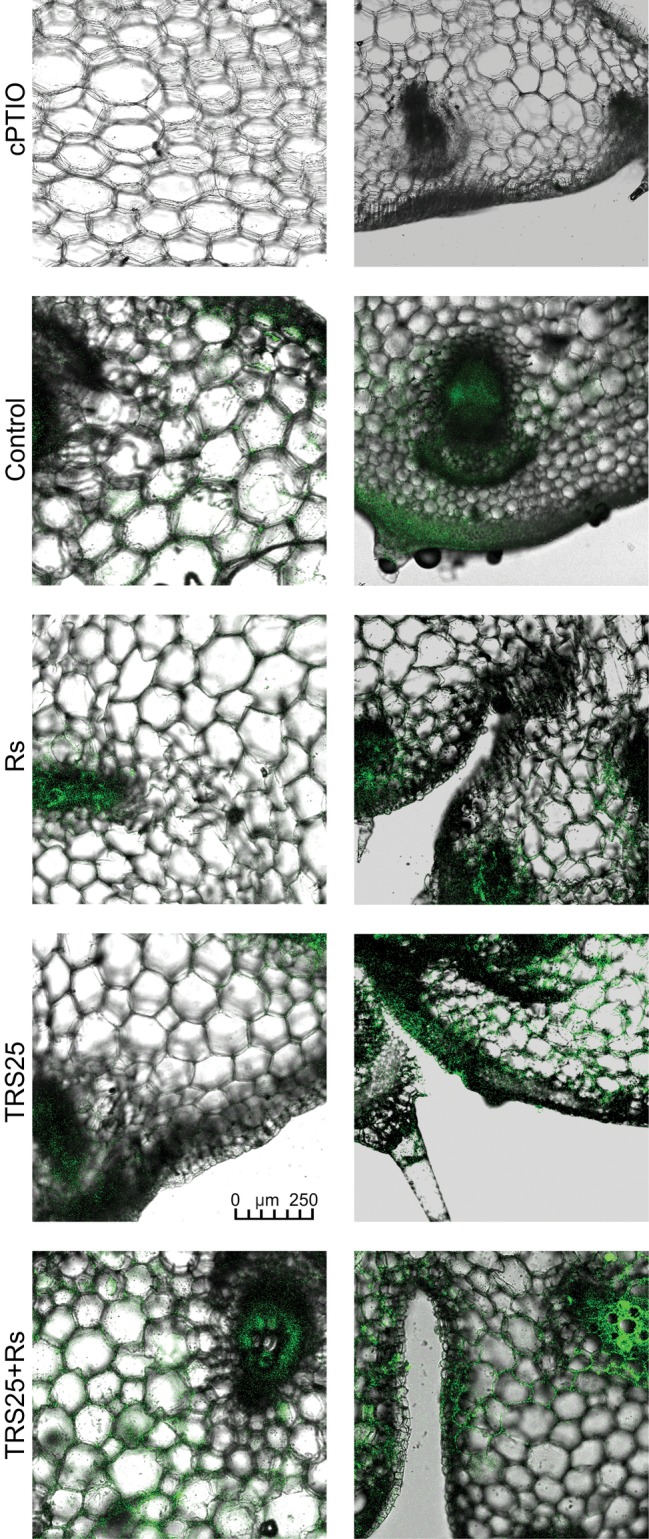
Cross sections of the cucumber shoots. Fluorescent confocal microscopy was used to detect NO localization in the cucumber plants. Light green fluorescence of NO was observed mainly in the cells localized in the vascular bundles, in the cells of epidermal tissues as well as in the area of plant trichome formation. As negative control, leaf pieces were immersed in a buffer containing cPTIO which eliminates NO. Abbreviations are as in Figure 1.

*R. solani* inoculation did not affect NO or SNO contents significantly in Rs plants as compared to the control (Figures 4A,B). Increased, by above 50%, NO content was observed in TRS25 and TRS25 + Rs plants as compared to the control (Figure 4A). No significant differences concerning NO content were observed between TRS25, TRS25 + Rs, and Rs plants. Enhanced SNO content was detected only in TRS25 + Rs plants as compared to the control, while no significant differences were detected for this parameter between TRS25 and TRS25 + Rs variants (Figure 4B). The microscopic analysis showed that in TRS25 and TRS25 + Rs plants, stronger fluorescence signals of NO were detected as compared to the control and Rs plants. In all tested plants, NO accumulation was observed in the cucumber leaves (Figure 5) and shoots (Figure 6), where the compound was accumulated as punctate distribution patterns mainly in the cells localized in the vascular bundles but also in the cells of epidermal tissues. Moreover, enhanced accumulation of NO was observed in the tissues localized in the area of plant trichome formation.

#### GSNOR Activity

The highest GSNOR activity was observed in Rs plants. It was significantly higher as compared to the control, TRS25, and TRS25 + Rs plants (Figure 4C). The strongest, by above 40%, GSNOR activity increase was observed in Rs plants as compared to TRS25 + Rs ones. On the other hand, there were no differences in the enzyme activity between TRS25 and control plants. Whereas, in TRS25 + Rs plants, GSNOR activity was the lowest, and significantly lower as compared to the control, Rs, and TRS25 plants (Figure 4C).

#### Salicylates Content

**Figure 7:**
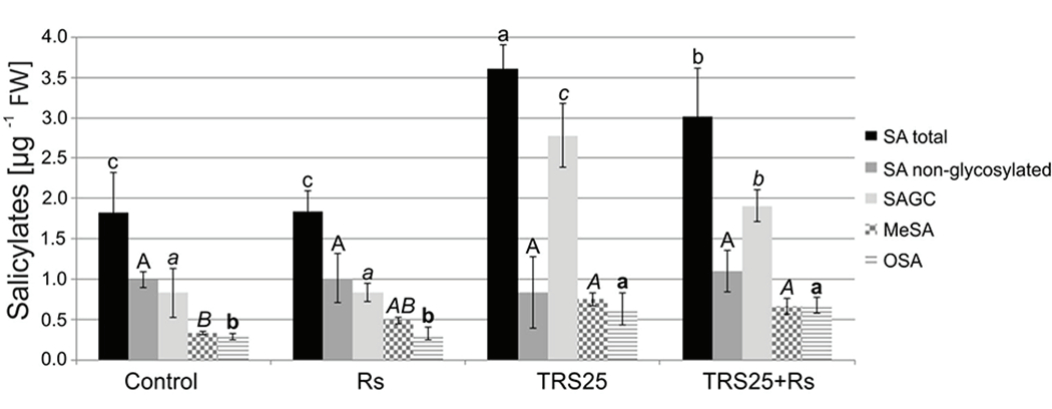
Effect of TRS25 on the content of salicylates including: total salicylic acid (total SA), non-glycosylated SA, SA glycosylated conjugates (SAGC), volatile methyl salicylate (MeSA), and octyl salicylate (OSA). The values represent the means + SE from five independent experiments with three replicates per variant. Statistical analysis of variance (ANOVA, *p* < 0.05, Duncan multiple range *post hoc* test) performed separately for salicylates demonstrate significant differences at *p* ≤ 0.05 between variants marked by letters a, b, and c for total SA; A for non-glycosylated SA; *a*, *b*, and *c* for SAGC; *A* and *B* for MeSA; and a and b for OSA.

As compared to the control, *R. solani* did not affect the content of salicylates in Rs plants, while pretreatment with TRS25 significantly increased their concentrations both in TRS25 and TRS25 + Rs plants (Figure 7). The strongest, above twofold, increase in the total SA was observed in TRS25 plants as compared to the control and Rs plants, and it was significantly higher as compared to TRS25 + Rs plants as well. Among all tested salicylates, TRS25 significantly increased SAGC accumulation, but did not trigger any significant changes in non-glycosylated SA in TRS25 and TRS25 + Rs plants as compared to the control and Rs plants. The highest, above threefold, increase in the content of SAGC was observed in TRS25 plants as compared to the control and Rs plants. In TRS25 + Rs plants, the increase in SAGC content was lower, almost twofold as compared to the control and Rs plants, and statistically lower as compared to TRS25 plants. The increase in SAGC accumulation observed in TRS25 and TRS25 + Rs plants was accompanied by increases in the content of volatile MeSA as compared to the control, and volatile OSA as compared to the control and Rs plants. The increases in both volatile compounds in TRS25 plants did not differ from those observed in TRS25 + Rs plants.

## Discussion

In this study, we present results which show that *T. atroviride* TRS25 is BCA, which significantly limits the development of infection and disease caused by the pathogenic fungus *R. solani* in cucumber plants (*C. sativus* L.). In the plants not pretreated with TRS25, *R. solani* caused progression of disease symptoms in roots and then in aboveground organs which had no direct contact with the pathogen. The observed disease symptoms were similar to those described in different plants including rice, tomato and cucumber, such as root rot, foliar blight, and damping-off (Singh et al., 2002; Bartz et al., 2010; Yousef et al., 2013; Jaiswal et al., 2015). Pretreatment of plants with *T. atroviride* TRS25 significantly reduced DI in cucumber plants. TRS25 delayed and limited disease symptoms in TRS25 + Rs roots and leaves as compared to the spread of the disease in Rs plants. In the previous studies, we showed that suppression of systemic disease symptoms observed on aboveground parts of the cucumber plants was not the result of direct influence of TRS25, as spores of this strain, similarly to *R. solani* were detected neither on, nor in cucumber shoots or leaves (Nawrocka et al., 2011; Szczech et al., 2014). Therefore, direct suppression of *R. solani* by secondary metabolites released by *Trichoderma*, implicated in other pathosystems (Howell, 2003; Contreras-Cornejo et al., 2009; Tucci et al., 2011), in the case of TRS25, might limit the disease, only to a certain extent. Taking into account our previous results which showed moderate ability of TRS25 to suppress directly *R. solani* development, the induction of systemic defense responses, as the main way of cucumber plant protection against this pathogen, was considered. Our results indicated that suppression of systemic disease symptoms in TRS25 + Rs plants might be related to the activation of cucumber defense responses by a complex signaling network, as observed previously in other plants protected by different *Trichoderma* strains, i.e., in cucumber protected against *Pseudomonas syringae* (Yedidia et al., 2003), tomato and onion against *Fusarium oxysporum* (Ozbay et al., 2004; Abdelrahman et al., 2016), or soybean against *Sclerotinia sclerotiorum* (Zhang et al., 2016).

In the present study, the obtained results pointed to biochemical changes, not observed in the defense signaling induced by *Trichoderma* in plants until now, effectively protecting them against *R. solani*. In the complex signaling network of defense responses, we observed significant changes in the contents of ROS, RNS, SNO, and salicylates, molecules important in this network, together with changes in the activity of enzymes involved in their metabolism including SOD, CAT, and GSNOR.

ROS, especially H_2_O_2_, and RNS with the dominance of NO, natural products of oxygen and nitrogen metabolism, are crucial signaling molecules involved in a wide range of physiological processes in plants. While ROS are permanently produced in chloroplasts, mitochondria, and peroxisomes as by-products of plant metabolism or as products of action of some enzymes with peroxidase or oxidase activity, the origin of NO in plants remains unclear. The number of non-enzymatic mechanisms together with numerous enzymatic sources of NO synthesis have been suggested in plants but only nitrate reductase was clearly identified as an NO-producing enzyme in land plants so far (Foyer and Noctor, 2009; Gupta et al., 2011; Scheler et al., 2013; del Río, 2015; Santolini et al., 2017). H_2_O_2_ and NO were shown to be rapidly generated in plants following challenge with biotrophic and necrotrophic pathogens. Increasing lines of evidence suggested their pivotal role during the development of plant defense responses and resistance (Mur et al., 2013; Scheler et al., 2013). It was previously documented that in the presence of *Trichoderma* spp. during defense responses induction, plants modulated their oxidation–reduction homeostasis by controlling generation and scavenging of ROS. Enhanced activity of CAT, SOD as well as ascorbate or glutathione peroxidases (APX, GPX), involved in ROS metabolism, was observed, for instance, in roots of sunflower plants protected by *Trichoderma harzianum* against *R. solani* (Singh et al., 2011), while H_2_O_2_ accumulation in *Arabidopsis thaliana* colonized by *Trichoderma virens* and *T. atroviride* was noted by Contreras-Cornejo et al. (2011) and Salas-Marina et al. (2011). It seems that H_2_O_2_ may play an important role in the signaling pathway of induced systemic defense responses. However, even though it is emphasized that its role as a signaling molecule is closely connected with NO (Scheler et al., 2013), no literature describing the interaction of these compounds during defense responses and plant protection induced by *Trichoderma* is available. In the tested plant-microbial system, it was shown that TRS25 significantly increased H_2_O_2_ and additionally NO contents, above all, in TRS25 + Rs plants with strongly reduced DI as compared to Rs plants. On the other hand, *Trichoderma* pretreatment did not affect $O_{2}^{.-}$ content, and further, where there was no excess of $O_{2}^{.-}$, it seemed that there was no need to stimulate the activity of SOD which is involved in removing its excess (Foyer and Noctor, 2009). Interestingly both in TRS25 and TRS25 + Rs plants the increased H_2_O_2_ content was accompanied by decrease in the activity of another antioxidant enzyme, CAT, involved in its conversion to H_2_O and O_2_. In this case, our results suggest that in plants protected against the pathogen by *Trichoderma* a rearrangement of the production and turnover of ROS and RNS occurred, regulating their bioavailability toward the accumulation of their most stable forms, that is H_2_O_2_ and NO. According to the suggestion of Corpas and Barroso (2015) NO might cooperate with H_2_O_2_ in the maintenance of cell oxidative-reduction homeostasis and in reduction of other toxic ROS generation. The use of $O_{2}^{.-}$, where there is excess of NO, to form other compounds, including peroxynitrite (ONOO^−^), can be an example (Rinalducci et al., 2008; Scheler et al., 2013; Corpas and Barroso, 2015). According to the previous reports, changes observed in cucumber plants treated with TRS25 might provide favorable conditions for H_2_O_2_ and NO to play the role of substrates or modulators of defense enzymes involved in plant protection against pathogens and, above all, of resistance signaling molecules (Djébali et al., 2011; Harman et al., 2012). These compounds transported systemically from cell to cell as well as in the case of NO through the vascular system in shoots and leaves of cucumber plants might take part in modulation of expression of genes involved in plant protection against diseases or they might regulate phytohormones involved in the signaling of resistance. Such suggestions were put forward, e.g., by Tada et al. (2008) and Scheler et al. (2013) who extensively described ROS and RNS crosstalk during biotic interactions of plants.

ROS and RNS were presented as important molecules which influence synthesis and activities of proteins crucial for plant protection against pathogens. According to the suggestion of del Río (2015) and Leitner et al. (2009), both groups of compounds may be involved in posttranslational modifications of proteins; however, the role and range of NO influence may be dominant. In this case, significant increase in NO observed in TRS25-treated plants should be emphasized. There are many ways by which NO might modulate the activity of defense related proteins including several main processes, that is, nitration, sulfenylation, and S-nitrosylation of proteins in the presence of ROS including H_2_O_2_ (Scheler et al., 2013; Corpas and Barroso, 2015; Wang et al., 2018). As a consequence, the mentioned transformations of the protein structures together with redox changes caused by ROS and RNS might enhance the activity of enzymes important in plant protection against different pathogens (Rinalducci et al., 2008; Leitner et al., 2009; Corpas and Barroso, 2015). Among them, S-nitrosylation was presented as an important process during plant defense responses against different pathogens. As described by Romero-Puertas et al. (2008), it played the key role in the modification of several proteins during the development of defense responses in a model of *A. thaliana* seedlings exposed to pathogenic infection by *P. syringae*. In the present study, involvement of S-nitrosylation in the *Trichoderma*-induced cucumber defense against *R. solani* may be also suggested. There are several explanations for the role of SNO, the accumulation of which was observed in TRS25 + Rs plants. Basically, when there is decrease in the activity of GSNOR, an enzyme involved in a reversible process of SNO reduction to glutathione disulphide and ammonia (Lee et al., 2008), they might be considered as important reservoirs of NO (Rinalducci et al., 2008; Corpas and Barroso, 2015). Secondly, S-nitrosylation might modulate activities of different enzymes involved in ROS and RNS metabolism, including CAT and GSNOR (Palmieri et al., 2010; Astier and Lindermayr, 2012; Ortega-Galisteo et al., 2012). Interestingly, despite the suggestion that enhanced activity of GSNOR is necessary for effective defense induction against different pathogens and that its mutation may disable plant defense responses and compromise resistance (Feechan et al., 2005), in the present study we did not observe suppressive influence of decreased activity of GSNOR and enhanced SNO formation on protection of cucumber plants against *R. solani*. Moreover, in TRS25 + Rs plants, downregulation of GSNOR and accumulation of SNO seemed to be effective ways to maintain enhanced content of active NO at a level that was safe for cucumber plants. In this context, NO might be linked with the other activators of defense genes including phytohormones, crucial for induction of plant protection (Mur et al., 2008).

Recently, it was documented that resistance induced by *Trichoderma* against different pathogens may involve a complex network of signaling molecules, including phytohormones such as SA, JA, and ET with their derivatives (Yedidia et al., 2003; Salas-Marina et al., 2011; Tucci et al., 2011; Martínez-Medina et al., 2013). Depending on the pathosystem, different relationships between phytohormones were observed in plants protected by *Trichoderma*. Basically, most studies demonstrated the important role of JA/ET-induced pathway protecting *Trichoderma*-treated plants against different pathogens. Systemic defense responses and resistance largely dependent on JA were observed, for example, in *A. thaliana* protected by *Trichoderma* against *Botrytis cinerea* (Contreras-Cornejo et al., 2011) and in tomato plants protected by *Trichoderma viride* against *F. oxysporum* or *R. solani* (Hafez et al., 2013), while *Trichoderma*-induced resistance in *A. thaliana* roots based on JA and ET signaling effectively protected them against *P. syringae* pv. *lachrymans* (Mathys et al., 2012). There is much less evidence suggesting an important role of combined simultaneous or time-shifted participation of JA and SA, and even less frequently SA alone, in plant resistance induced by *Trichoderma*. Nevertheless, TISR characterized by upregulation of signaling pathways dependent on JA and SA was presented, for instance, in cucumber plants protected by *T. asperellum* against *P. syringae* pv. *lachrymans* (Segarra et al., 2007), in potato plants protected by *T. harzianum* against *R. solani* (Gallou et al., 2009) and in tomato plants protected by *T. virens* against *Fusarium oxysporum* (Jogaiah et al., 2018). In the present study, TRS25 pretreatment significantly reduced systemic spread of *R. solani*-induced disease and simultaneously increased content of SA with its derivatives. Moreover, accumulation of this phytohormone was observed in the plants only treated with *Trichoderma*. Inversely, Rs plants showed no significant changes in SA content. Interestingly, in the previous studies, the tested TRS25 strongly stimulated accumulation of hydroxybenzoic acids being precursors of SA as well as other compounds included in the group of salicylates and phenolics (Nawrocka et al., 2017). On the other hand, neither TRS25 nor *R. solani* influenced the content of JA, its derivatives and ET in cucumber plants, while they significantly increased the generation of other unsaturated fatty acid derivatives including Z-3-hexanal, Z-3-hexenol, and E-2-hexenal, which might play the role of signaling molecules as well (Nawrocka et al., 2017, 2018). The present results indicated that the systemic defense responses essential to decrease disease incidence, observed in TRS25 + Rs plants, largely depended on SA biosynthesis. This is consistent with the previously described observations concerning plants protected by *T. atroviride* and *T. harzianum* against *B. cinerea* (Tucci et al., 2011) and *A. thaliana* protected by *T. asperellum* against *Cucumber mosaic virus* (Elsharkawy et al., 2013), where SA played the crucial role in upregulation of defense genes and active resistance mechanisms. In the present study, we assessed involvement of different derivatives of SA in defense induced against *R. solani*. The tested TRS25 strain significantly enhanced accumulation of volatile MeSA and OSA, molecules which might be involved in long distance signaling characteristic of SAR (Silverman et al., 2005; Park et al., 2007; Gao et al., 2014). According to Scala et al. (2013), volatile compounds including SA derivatives might be transported faster than signal molecules from cell to cell or through the vascular system, and might act synergistically to ensure optimal protection in distal plant parts. The complex network of defense inducers including SA derivatives and unsaturated fatty acid derivatives, accumulated in TRS25 + Rs plants, may explain the enhanced expression of genes, including *PR1*, *PR4* and *PR5*, characteristic of two kinds of systemic resistance, i.e., ISR/SAR, observed previously in cucumber plants treated with *T. atroviride* TRS25 (Nawrocka et al., 2018). Moreover, in TRS25 and TRS25 + Rs plants the accumulated SAGC might be a reservoir of SA releasing it when necessary (Takatsuji, 2014). In this case, through conjugation with small organic molecules such as glucose, an inactive form of SA could be transported into and stored in plant vacuoles as it was observed in *A. thaliana* (Thompson et al., 2017).

In the present study, the important role of SA, NO and H_2_O_2_ as signaling molecules of cucumber systemic defense responses activated by TRS25 against *R. solani* is suggested. In the tested pathosystem containing *T. atroviride* TRS25, possible cooperation of the mentioned molecules, which may be an important element of complicated signaling of defense responses induced by *Trichoderma*, is described for the first time. However, based on the results of studies using other experimental systems, several hypothetical modes of action of signaling molecules may be taken into account. Together with other processes, including redox-based SNO formation and glucosylation of SA, all the accumulated compounds might influence mutually their contents and activities (Astier and Lindermayr, 2012). First of all, in the present study, NO might be linked with the accumulation of SA, but not with the accumulation of JA and ET, cooperating in transcriptional activation of defense genes as suggested by Mur et al. (2008). As a substrate in S-nitrosylation, it might participate, for instance, in inhibition of activities of enzymes involved in H_2_O_2_ and SA removal, including CAT (Astier and Lindermayr, 2012). Moreover, accumulation of H_2_O_2_ might be also the result of inactivation of the same enzyme by SA binding (Klessig et al., 2000). In turn, excessive release of NO might be controlled by formation of SNO with simultaneous decrease in SOD and GSNOR activities involved in SNO catabolism and release of free, active NO (Gaston et al., 2003; Rustérucci et al., 2007). A similar correlation was observed in *A. thaliana*, where downregulation of the GSNOR gene induced remarkable increase in SNO content, improved SAR, and consequently enhanced effective protection against *Peronospora parasitica* (Lee et al., 2008; Moreau et al., 2010). On the other hand, as it was uncovered in other pathosystems, changes in the cellular SNO concentrations might regulate both accumulation of SA, the plant immune activator and expression of SA-dependent genes (Feechan et al., 2005; Loake and Grant, 2007). Therefore, the obtained results may suggest that NO *via* S-nitrosylation might be one of the key regulators of SA-dependent systemic defense responses in TRS25-treated plants. In the present context, SA is proposed to play an important role in the systemic defense responses activated by TRS25 in cucumber plants, effectively protecting them against *R. solani-*induced disease. This phytohormone itself or by increasing the content of H_2_O_2_ or NO could trigger formation of active NPR1 proteins, responsible for regulating the expression of genes that code plant defense proteins. NPR1 present as oligomer in the cytoplasm, stabilized by intermolecular disulfide bonds is susceptible to changes in redox state. Accumulated SA and H_2_O_2_, by changing the state of the cell reduction, could reduce disulfide bonds, causing NPR1 degradation into monomers which, moved into a nucleus, might consequently activate expression of genes related to pathogenesis, necessary to the development of systemic resistance (Klessig et al., 2000; Tada et al., 2008; Mathys et al., 2012; del Río, 2015). It is highly possible that the mentioned compounds may be accompanied by other signaling molecules including volatiles; however, additional studies are necessary to determine them.

The present study clearly indicated that the *T. atroviride* TRS25 strain significantly reduced *R. solani* infection in cucumber plants. Treatment of the cucumber roots with TRS25 induced oxidative, signaling, and biochemical changes, very important during induction of plant defense responses. In the tested plant-microbial system including TRS25 we suggest, for the first time, cooperation of redox-active small molecules including H_2_O_2_ or NO, creating so called oxidative signaling, with SA phytohormone and its derivatives including MeSA and OSA. We suggest that in TRS25 and TRS25 + Rs plants, the accumulation of H_2_O_2_ and NO might be as a result of CAT and GSNOR activity decrease. On the other hand, excessive accumulation of NO and SA might be controlled by forming their inactive forms, SNO and SAGC, respectively. We suggest that all the mentioned compounds may play an important role in the complex signaling network positively involved in the protection of cucumber plants against *R. solani* activated by TRS25.

## Author Contributions

JN and UM conceived the idea and wrote the paper. JN, AG, and UM performed the analyses and interpreted the results.

### Conflict of Interest Statement

The authors declare that the research was conducted in the absence of any commercial or financial relationships that could be construed as a potential conflict of interest.
